# Supplementary material for: Polycomb contraction differentially regulates terminal human hematopoietic differentiation programs
Source: BMC Biol. 2022 May 13;20:104. doi: 10.1186/s12915-022-01315-1 (PMC9102747; doi:10.1186/s12915-022-01315-1)
Supplement: Supplementary file 1 — Additional file 1: Fig. S1 to 8. Fig S1 - Sorting strategy for hematopoietic populations profiled in this study. Fig S2 - Progenitor populations possess unique expression profiles. Fig S3 - H3K27me3 promoter density signatures are conserved across progenitor populations. Fig S4 - H3K27me3 LOCKs lost during myeloid differentiation are associated with LADs in CD34+ progenitor cells. Fig S5 - BMI1 loss reduces genome-wide levels of H3K27me3. Fig S6 - Enhancers dynamics in erythroid precursor, monocyte and differentiated lymphoid cells. Fig S7 - Lineage-specific enhancers are marked by H3K27ac in hematopoietic progenitor subsets. Fig S8 - Lineage specific enhancers harbor lineage specific TF binding site are marked with H3K27ac in progenitor population. [file 12915_2022_1315_MOESM1_ESM.pdf]

Supplemental Figures and Legends

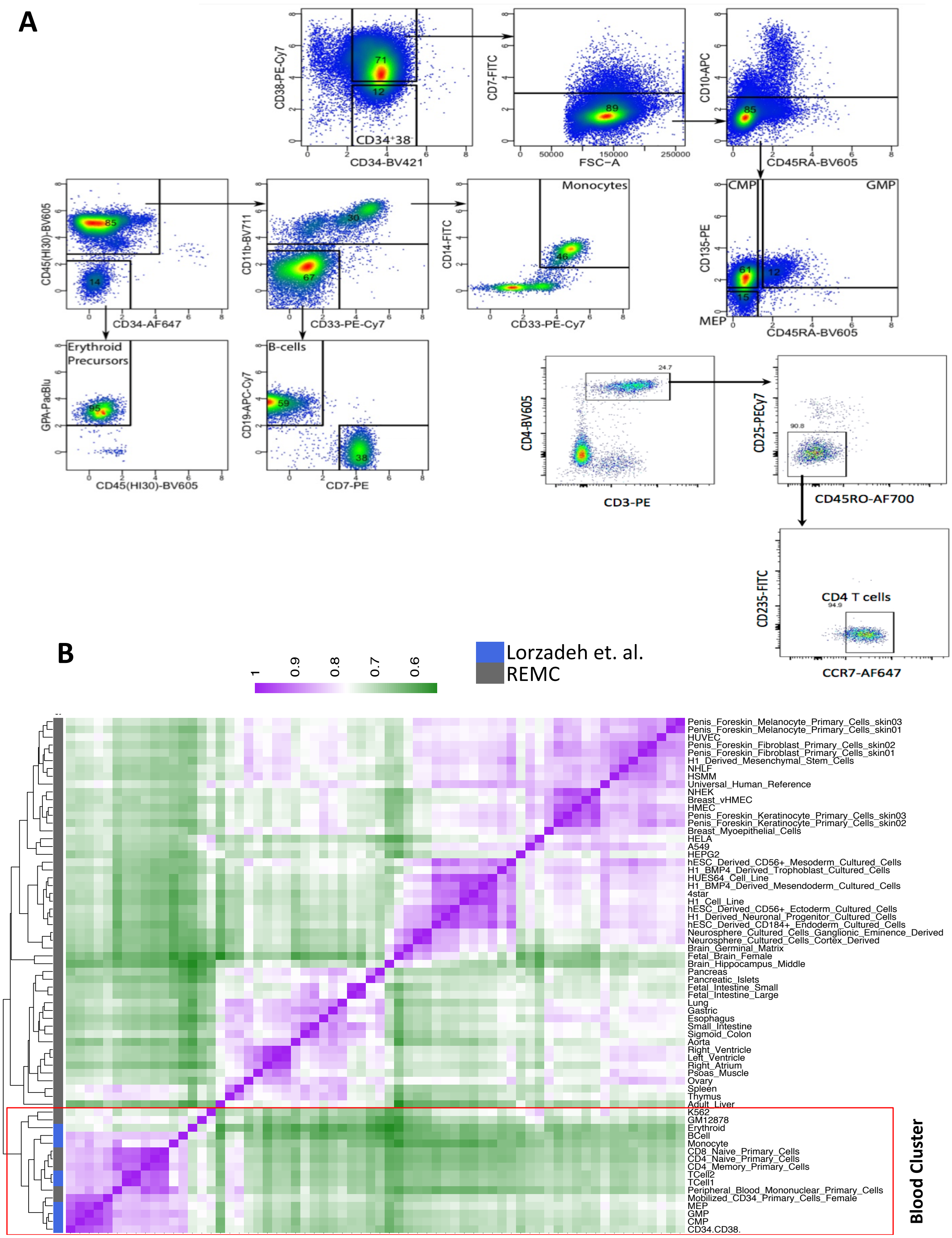

Figure S1. Sorting strategy for hematopoietic populations profiled in this study. A) Representative examples of sorting strategies for CD34+CD38-, CMP, GMP, MEP, monocytes, erythroblasts, B and T cell isolated from cord blood pools. **B)** Unsupervised hierarchical clustering and heatmap of pairwise spearman correlations for protein coding gene RPKM values across blood cell types profiled in this study in the context of all cell types profiled by NIH Epigenome RoadMap Consortium. The cluster of blood cell types is indicated by the shaded box.

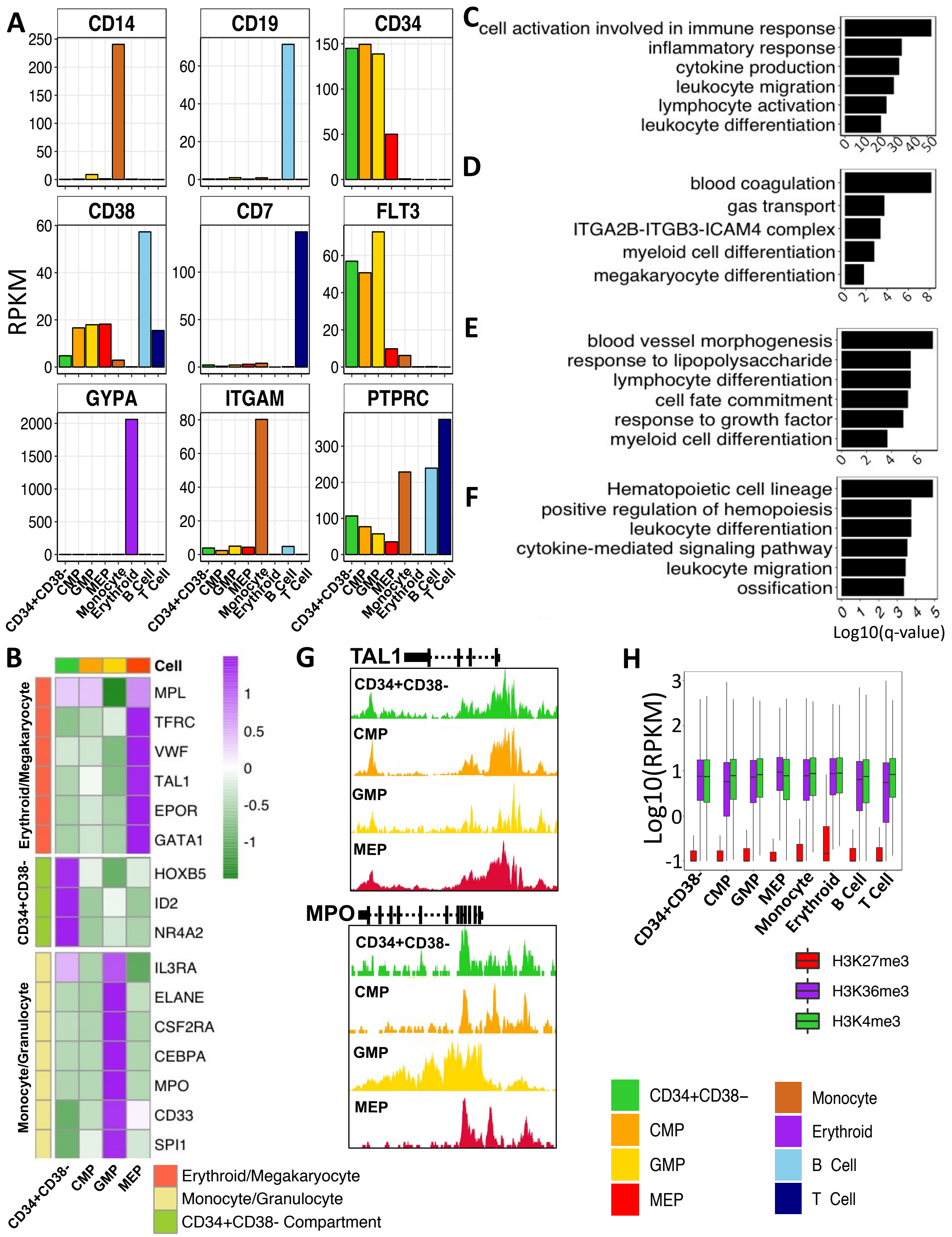
A
RPKM
CD14
CD19
CD34
CD38
CD7
FLT3
GYPA
ITGAM
PTPRC
CD34+CD38-
CMP
GMP
MEP
Monocyte
Erythroid
B Cell
T Cell
C
cell activation involved in immune response
inflammatory response
cytokine production
leukocyte migration
lymphocyte activation
leukocyte differentiation
D
blood coagulation
gas transport
ITGA2B-ITGB3-ICAM4 complex
myeloid cell differentiation
megakaryocyte differentiation
E
blood vessel morphogenesis
response to lipopolysaccharide
lymphocyte differentiation
cell fate commitment
response to growth factor
myeloid cell differentiation
F
Hematopoietic cell lineage
positive regulation of hemopoiesis
leukocyte differentiation
cytokine-mediated signaling pathway
leukocyte migration
ossification
Log10(q-value)
B
Cell
Erythroid/Megakaryocyte
CD34+CD38-
Monocyte/Granulocyte
MPL
TFRC
VWF
TAL1
EPOR
GATA1
HOXB5
ID2
NR4A2
IL3RA
ELANE
CSF2RA
CEBPA
MPO
CD33
SPI1
Erythroid/Megakaryocyte
Monocyte/Granulocyte
CD34+CD38- Compartment
G
TAL1
MPO
CD34+CD38-
CMP
GMP
MEP
H
Log10(RPKM)
H3K27me3
H3K36me3
H3K4me3
CD34+CD38−
CMP
GMP
MEP
Monocyte
Erythroid
B Cell
T Cell

Figure S2. Progenitor populations possess unique expression profiles. A) Expression (RPKM) of cell type specific cell surface marker genes across cell types as indicated by the colour legend on the bottom right. **B)** Expression of previously identified progenitor population specific genes across CD34+CD38-, CMP, GMP and MEP. Pathway enrichment analysis of GMP **(C)**, MEP **(D)**, CD34+CD38- **(E)**, and CMP **(F)** differentially expressed gene sets identified by DEFine (FDR > 0.01). **G)** Genome browser view of H3K4me3 density in progenitor populations at the *TAL1* and *MPO* locus across CD34+CD38-, CMP, GMP and MEP. **H)** Expression of genes marked with H3K4me3, H3K27me3 or H3K36me3 across each cell type.

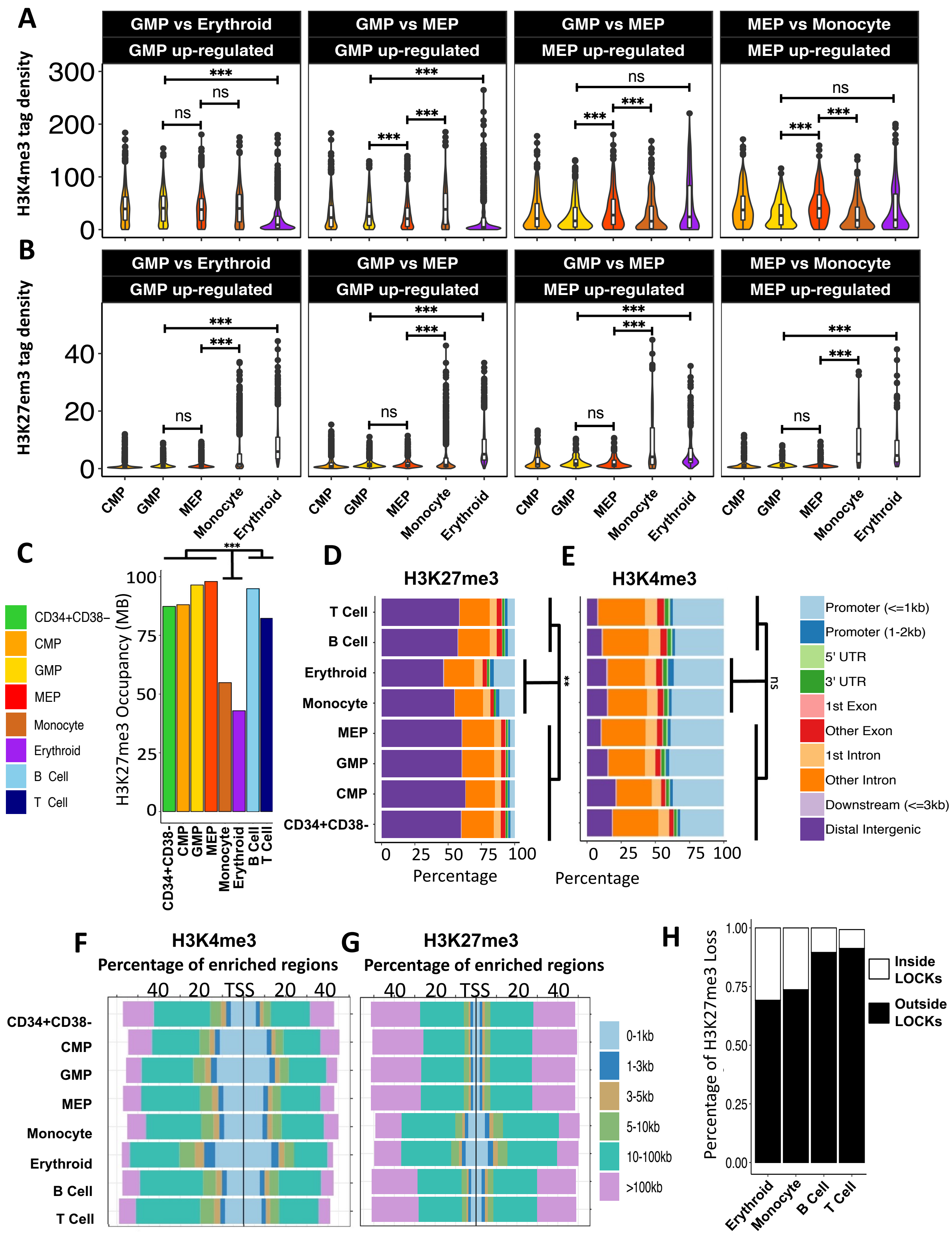

A
GMP vs Erythroid
GMP up-regulated
GMP vs MEP
GMP up-regulated
GMP vs MEP
MEP up-regulated
MEP vs Monocyte
MEP up-regulated
H3K4me3 tag density
B
H3K27em3 tag density
CMP
GMP
MEP
Monocyte
Erythroid
C
CD34+CD38−
CMP
GMP
MEP
Monocyte
Erythroid
B Cell
T Cell
H3K27me3 Occupancy (MB)
D
H3K27me3
E
H3K4me3
Percentage
Promoter (<=1kb)
Promoter (1-2kb)
5' UTR
3' UTR
1st Exon
Other Exon
1st Intron
Other Intron
Downstream (<=3kb)
Distal Intergenic
F
H3K4me3
Percentage of enriched regions
G
H3K27me3
Percentage of enriched regions
TSS
0-1kb
1-3kb
3-5kb
5-10kb
10-100kb
>100kb
H
Percentage of H3K27me3 Loss
Inside LOCKs
Outside LOCKs

Figure S3. H3K27me3 promoter density signatures are conserved across progenitor populations. H3K4me3 **(A)** and H3K27me3 **(B)** tag density ±2 Kb of transcription start sites of genes up-regulated in GMP and MEP across CMP, GMP, MEP, monocyte and erythroid precursor as indicated by the colour legend. **C)** Plot of the cumulative number of base pairs marked by H3K27me3 across cell types indicated by colour legend on the bottom right. Percentage of H3K27me3- **(D)** and H3K4me3-**(E)** enriched regions at the genomic features indicated. Percentage of H3K4me3 **(F)** and H3K27me3 **(G)** enriched regions with respect to their binned distance to the TSS of coding genes across cell types indicated by colours. Distance from TSS for each bin indicated in the bottom panel. **H)** Fraction of H3K27me3 loss in erythroid and monocyte within or outside the LOCKs (** $P < 0.01$, *** $P < 0.001$).

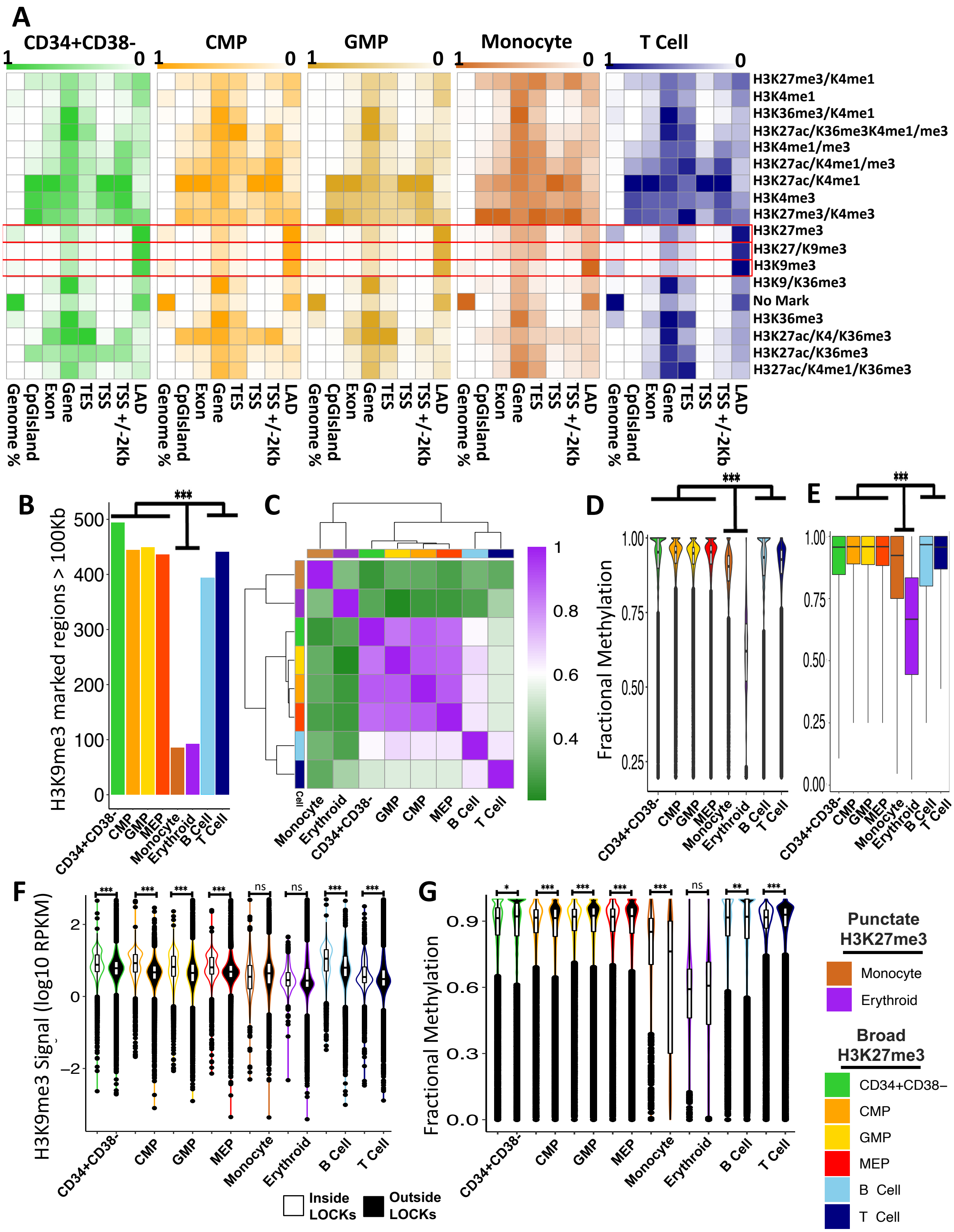
A
CD34+CD38-
CMP
GMP
Monocyte
T Cell
1
0
H3K27me3/K4me1
H3K4me1
H3K36me3/K4me1
H3K27ac/K36me3K4me1/me3
H3K4me1/me3
H3K27ac/K4me1/me3
H3K27ac/K4me1
H3K4me3
H3K27me3/K4me3
H3K27me3
H3K27/K9me3
H3K9me3
H3K9/K36me3
No Mark
H3K36me3
H3K27ac/K4/K36me3
H3K27ac/K36me3
H327ac/K4me1/K36me3
Genome %
CpGIsland
Exon
Gene
TES
TSS
TSS +/-2Kb
LAD
B
H3K9me3 marked regions > 100Kb
***
500
400
300
200
100
0
CD34+CD38-
CMP
GMP
MEP
Monocyte
Erythroid
B Cell
T Cell
C
1
0.8
0.6
0.4
Monocyte
Erythroid
CD34+CD38-
GMP
CMP
MEP
B Cell
T Cell
D
***
Fractional Methylation
1.00
0.75
0.50
0.25
E
***
1.00
0.75
0.50
0.25
0.00
F
H3K9me3 Signal (log10 RPKM)
***
ns
2
0
−2
Inside LOCKs
Outside LOCKs
G
Fractional Methylation
*
**
0.9
0.6
0.3
0.0
Punctate H3K27me3
Monocyte
Erythroid
Broad H3K27me3
CD34+CD38–
CMP
GMP
MEP
B Cell
T Cell

Figure 4. H3K27me3 LOCKs lost during myeloid differentiation are associated with LADs in CD34+ progenitor cells. A) Emission probabilities by histone mark for the 18 states of the ChromHMM chromatin state model across all cell types (upper panel). Emission probabilities are based on ChIP-seq data presented in this study. Enrichment of chromatin states within genomic features for MEPs (red), erythroblasts (purple) and B cells (blue). **B)** Cumulative number of bases marked by indicated histone modification within progenitor population LOCKs as indicated by the colour legend (bottom right). **C)** Genome browser view of H3K27me3 and H3K9me3 density on chromosome 20 across cell types indicated by the colour legend (bottom left). The shaded box indicates a H3K27me3 LOCK (FDR < 0.05) present in progenitor cells but lost in monocytes and erythroblasts. ChromHMM derived heatmap of z-score enrichment of identified polycomb repressed **(D)** and dually repressed **(E)** chromatin states at genomic features indicated. **F)** Barplot showing percentage of LOCKs overlapping with laminB binding sites. **G)** Barplot showing percentage overlap of LOCKs lost in monocyte or erythroblasts with laminB binding site. **H)** Violin plot showing occupancy of H3K9me3 (log10(bps)) at LOCKs across all populations as indicated by the colour legend (bottom right). Violin plot of fractional CpG methylation levels within progenitors' LOCKs **(I)**, H3K27me3 enriched regions **(J)** and regions containing both H3K27me3 and H3K9me3 **(K)** across all populations as indicated by the colour legend (bottom right) (*** $p < 0.001$).

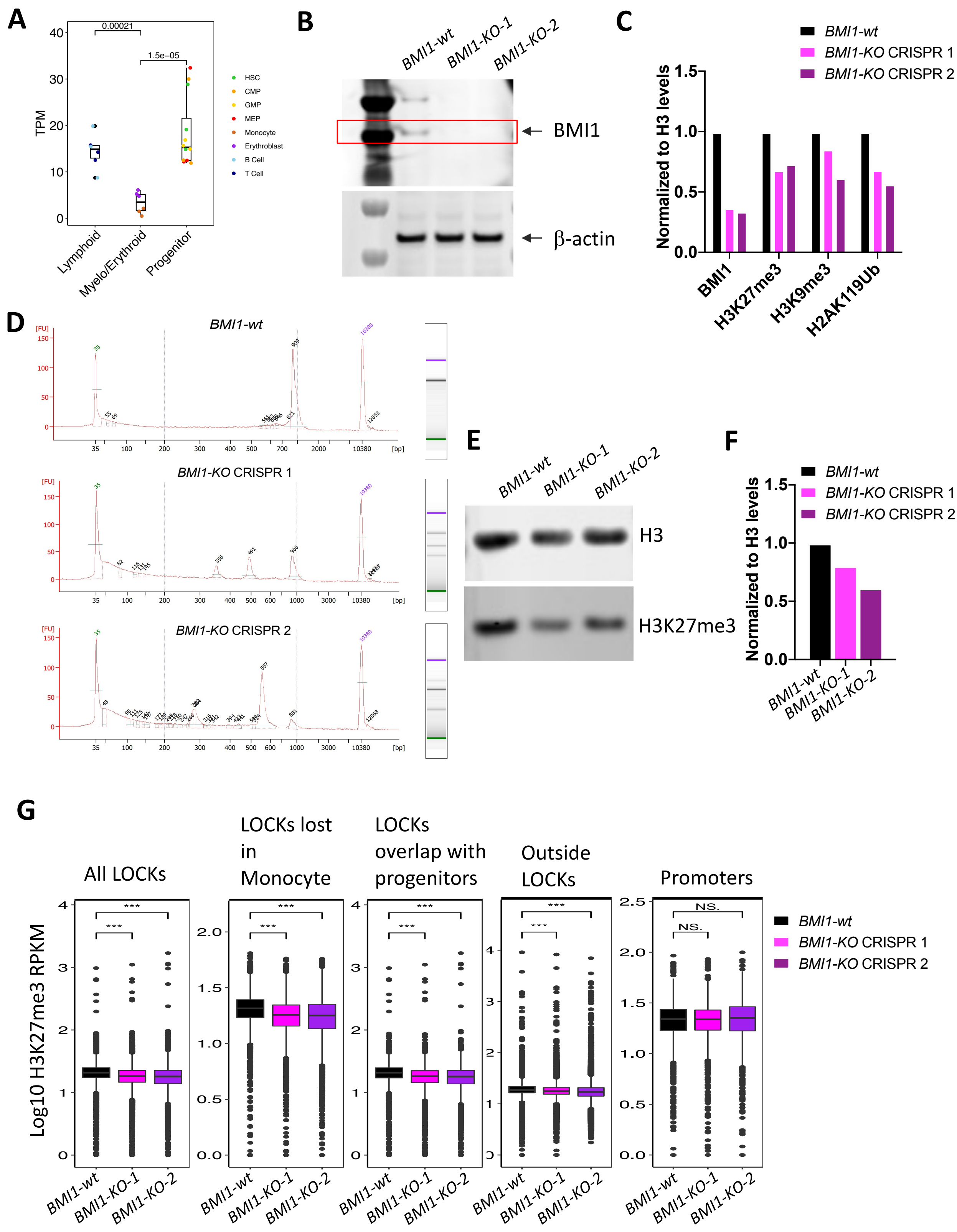
A
0.00021
1.5e-05
TPM
HSC
CMP
GMP
MEP
Monocyte
Erythroblast
B Cell
T Cell
Lymphoid
Myelo/Erythroid
Progenitor
B
BMI1-wt
BMI1-KO-1
BMI1-KO-2
BMI1
β-actin
C
BMI1-wt
BMI1-KO CRISPR 1
BMI1-KO CRISPR 2
Normalized to H3 levels
BMI1
H3K27me3
H3K9me3
H2AK119Ub
D
BMI1-wt
BMI1-KO CRISPR 1
BMI1-KO CRISPR 2
[FU]
[bp]
E
BMI1-wt
BMI1-KO-1
BMI1-KO-2
H3
H3K27me3
F
BMI1-wt
BMI1-KO CRISPR 1
BMI1-KO CRISPR 2
Normalized to H3 levels
G
All LOCKs
LOCKs lost in Monocyte
LOCKs overlap with progenitors
Outside LOCKs
Promoters
Log10 H3K27me3 RPKM
NS.
BMI1-wt
BMI1-KO CRISPR 1
BMI1-KO CRISPR 2

Figure S5. BMI1 loss reduces genome-wide levels of H3K27me3. A) BMI1 expression across hematopoietic cells. RNAseq acquired from Blueprint consortium. **B)** Global measurement of BMI1 and β-actin by western blot across *BMI1-wt* and *KO* (N=2). **C)** Quantitative measurement of western blot intensity shown in **Figure 5C** and **S5A** (N=2). **D)** Agilent bioanalyzer profile of PCR amplicon after T7E1 mutation cleavage assay in CRISPR Control (*BMI1-wt*) and *BMI1-KO* replicates. **E)** Global measurement of H3 and H3K27me3 by western blot in *BMI1-wt* and *KO* (N=2). **F)** Quantitative measurement of western blot intensity shown in **S5D** (N=2). **G)** H3K27me3 density at LOCKs identified in HL60 , those overlapped with primary CB progenitor LOCKs, those that are lost in monocyte across *BMI1-wt* and *KO*, at H3K27me3 enriched regions outside the LOCKs and at promoters (+/-2kb TSS). (* $p < 0.05$, ** $p < 0.01$ and *** $p < 0.001$).

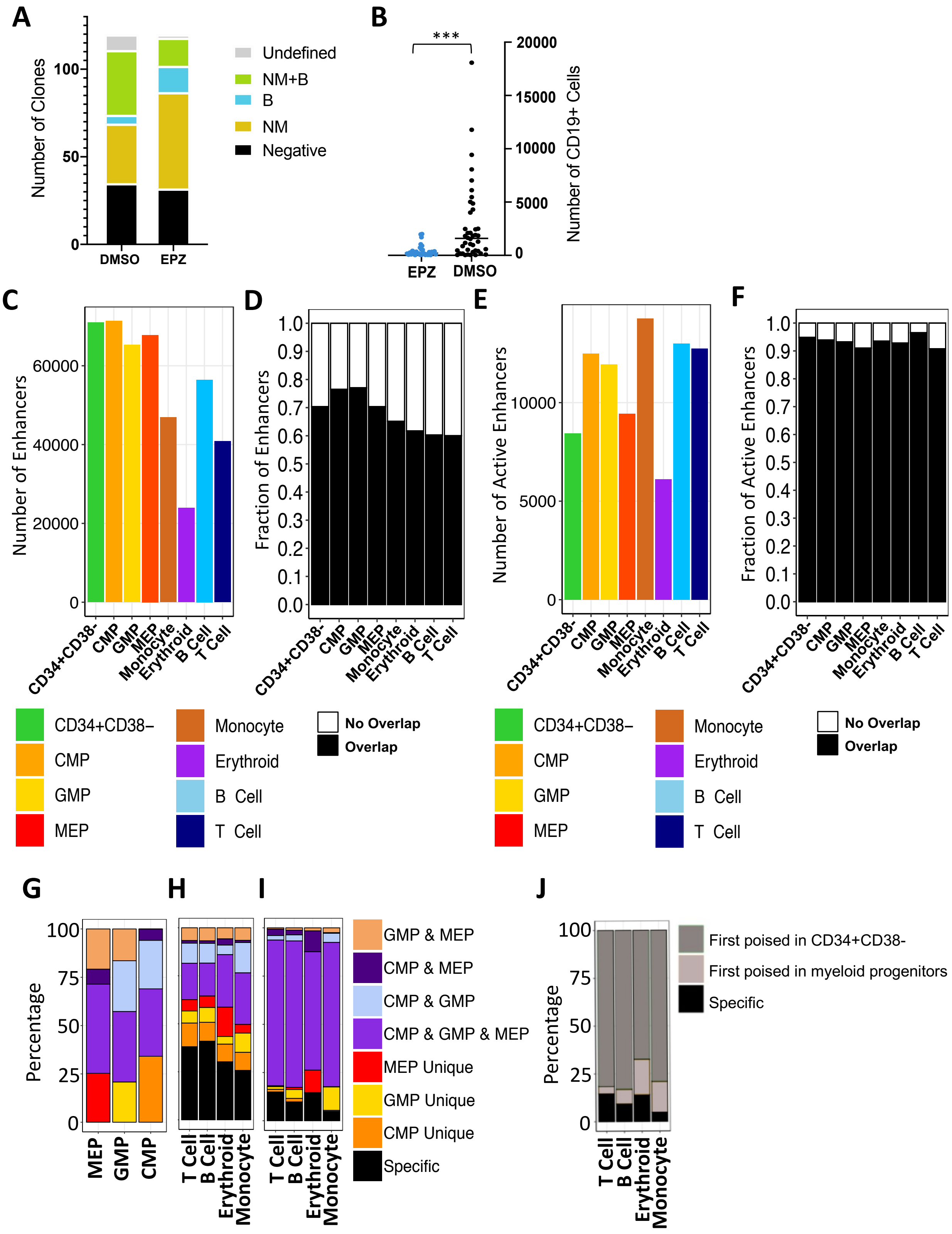
A
Number of Clones
0
50
100
DMSO
EPZ
Undefined
NM+B
B
NM
Negative
B
***
Number of CD19+ Cells
0
5000
10000
15000
20000
EPZ
DMSO
C
Number of Enhancers
0
20000
40000
60000
D
Fraction of Enhancers
0.0
0.1
0.2
0.3
0.4
0.5
0.6
0.7
0.8
0.9
1.0
E
Number of Active Enhancers
0
5000
10000
F
Fraction of Active Enhancers
CD34+CD38-
CMP
GMP
MEP
Monocyte
Erythroid
B Cell
T Cell
CD34+CD38–
CMP
GMP
MEP
Monocyte
Erythroid
B Cell
T Cell
No Overlap
Overlap
G
H
I
J
Percentage
0
25
50
75
100
MEP
GMP
CMP
T Cell
B Cell
Erythroid
Monocyte
GMP & MEP
CMP & MEP
CMP & GMP
CMP & GMP & MEP
MEP Unique
GMP Unique
CMP Unique
Specific
First poised in CD34+CD38-
First poised in myeloid progenitors
Specific

Figure S6. Enhancers dynamics in erythroid precursor, monocyte and differentiated lymphoid cells. A) Bar plot showing the number of clones with different contents. M = CD14+ monocytes, N= CD15+ neutrophils, B = CD19+ B cells, negative = <10 CD45+ events, undefined = no detectable mature cells. **B)** Total number of cells expressing CD19+.**C)** Number of enhancers (active and primed enhancers) across cell type. **D)** Fraction of Enhancers (active and primed enhancers) that overlap with open chromatin regions. **E)** Total number of active enhancers across cell types as indicated by the colour legend. **F)** Fraction of active Enhancers that overlap with open chromatin regions. **G)** Percentage of active enhancers that are shared between or unique to each progenitor populations. **H)** Percentage of active enhancers that are present in progenitor populations or *de novo* (specific) in differentiated cells as indicated by the colour legend. **I)** Percentage of active enhancers that are poised in progenitor populations or *de novo* in differentiated cells as indicated by the colour legends. **J)** Percentage of active enhancers present in differentiated cells that are first poised in CD34+CD38- cells, progenitor cells, or appear *de novo* in erythroid precursors, monocytes or lymphoid cells. Cell types are indicated by the colour legend. (* $p < 0.05$, ** $p < 0.01$ and *** $p < 0.001$)

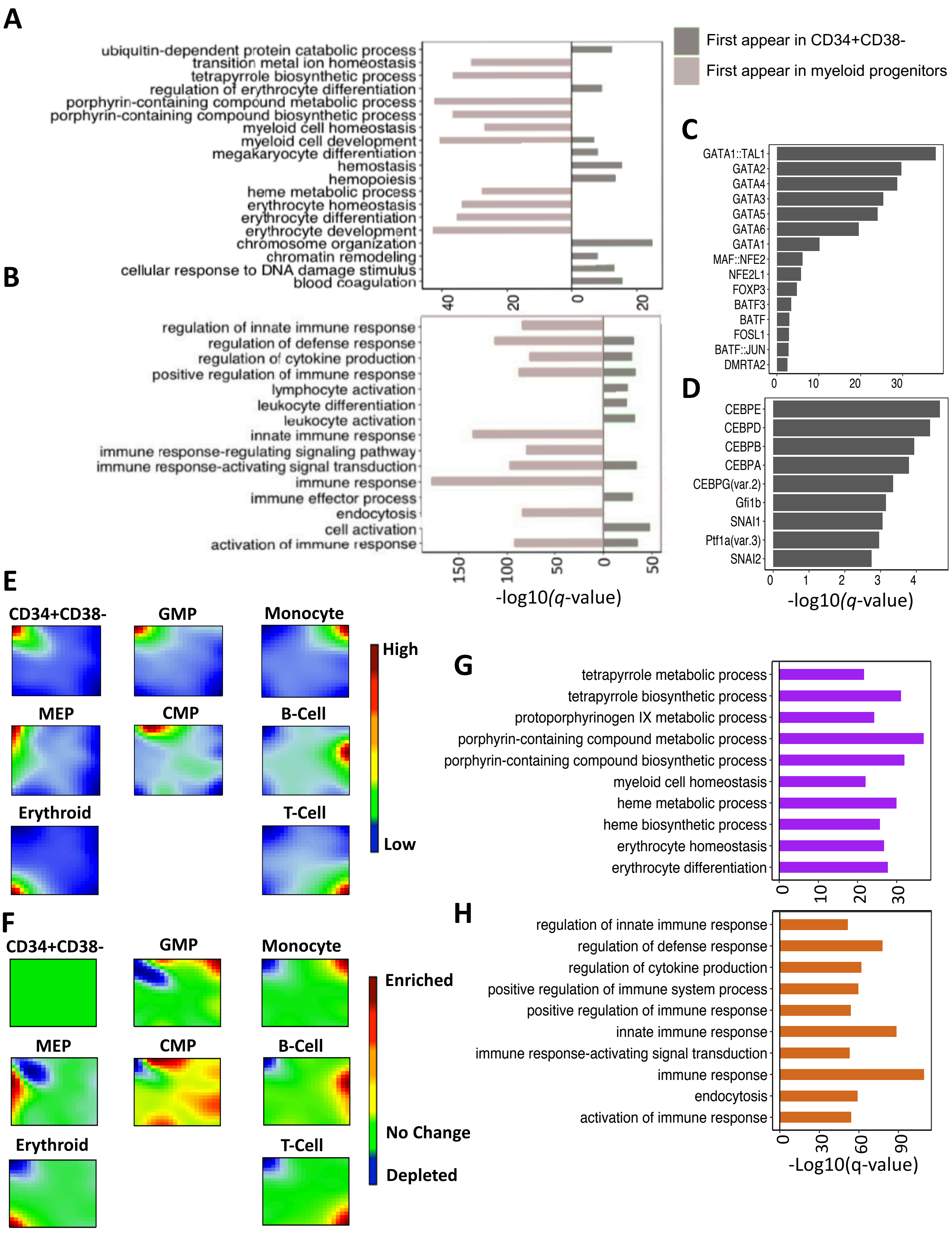
A
B
C
D
E
F
G
H
First appear in CD34+CD38-
First appear in myeloid progenitors
ubiquitin-dependent protein catabolic process
transition metal ion homeostasis
tetrapyrrole biosynthetic process
regulation of erythrocyte differentiation
porphyrin-containing compound metabolic process
porphyrin-containing compound biosynthetic process
myeloid cell homeostasis
myeloid cell development
megakaryocyte differentiation
hemostasis
hemopoiesis
heme metabolic process
erythrocyte homeostasis
erythrocyte differentiation
erythrocyte development
chromosome organization
chromatin remodeling
cellular response to DNA damage stimulus
blood coagulation
regulation of innate immune response
regulation of defense response
regulation of cytokine production
positive regulation of immune response
lymphocyte activation
leukocyte differentiation
leukocyte activation
innate immune response
immune response-regulating signaling pathway
immune response-activating signal transduction
immune response
immune effector process
endocytosis
cell activation
activation of immune response
-log10(q-value)
GATA1::TAL1
GATA2
GATA4
GATA3
GATA5
GATA6
GATA1
MAF::NFE2
NFE2L1
FOXP3
BATF3
BATF
FOSL1
BATF::JUN
DMRTA2
CEBPE
CEBPD
CEBPB
CEBPA
CEBPG(var.2)
Gfi1b
SNAI1
Ptf1a(var.3)
SNAI2
-log10(q-value)
CD34+CD38-
GMP
Monocyte
MEP
CMP
B-Cell
Erythroid
T-Cell
High
Low
Enriched
No Change
Depleted
tetrapyrrole metabolic process
tetrapyrrole biosynthetic process
protoporphyrinogen IX metabolic process
porphyrin-containing compound metabolic process
porphyrin-containing compound biosynthetic process
myeloid cell homeostasis
heme metabolic process
heme biosynthetic process
erythrocyte homeostasis
erythrocyte differentiation
regulation of innate immune response
regulation of defense response
regulation of cytokine production
positive regulation of immune system process
positive regulation of immune response
innate immune response
immune response-activating signal transduction
immune response
endocytosis
activation of immune response
-Log10(q-value)

Figure S7. Lineage-specific enhancers are marked by H3K27ac in hematopoietic progenitor subsets. GREAT pathway enrichment analysis of active enhancers in erythroid precursor (**A**) and monocytes (**B**) that are first apparent in $CD34^{+}CD38^{-}$ or other progenitor subsets. Transcription binding site enrichment at active enhancers in erythroblasts (**C**) and monocytes (**D**) that first appear in their myeloid progenitor populations with respect to those that first appear in CD34+CD38-. **E**) SOM plot of rank normalized H3K27ac signal contained within the hematopoietic enhancer catalogue across cell types profiled in this study. **F**) SOM plot of rank normalized H3K27ac signal within hematopoietic enhancers with respect to CD34+CD38- for each cell type. Gene ontology analysis of super enhancers in erythroid precursor (**G**) and monocyte (**H**).

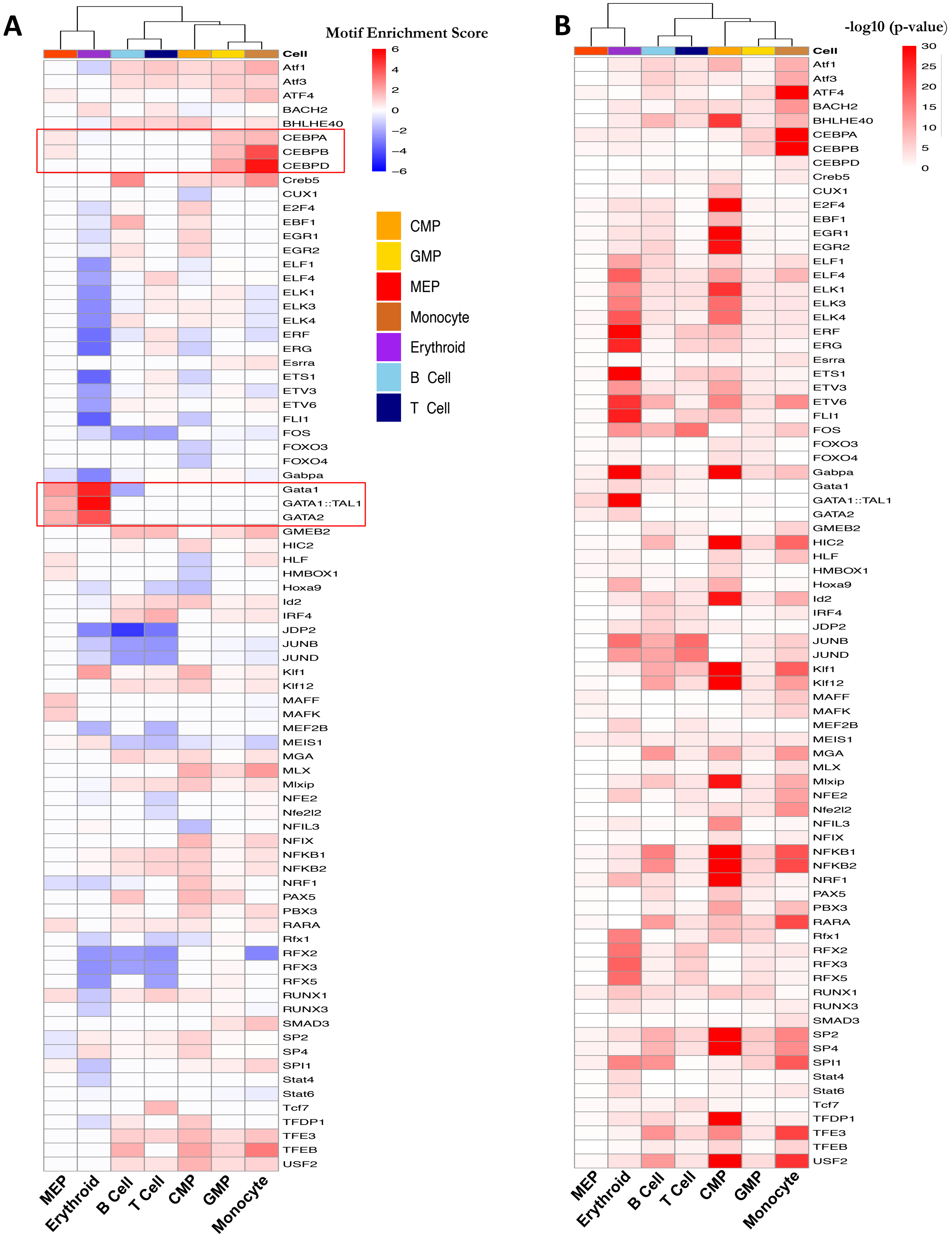
A
Motif Enrichment Score
6
4
2
0
−2
−4
−6
Cell
Atf1
Atf3
ATF4
BACH2
BHLHE40
CEBPA
CEBPB
CEBPD
Creb5
CUX1
E2F4
EBF1
EGR1
EGR2
ELF1
ELF4
ELK1
ELK3
ELK4
ERF
ERG
Esrra
ETS1
ETV3
ETV6
FLI1
FOS
FOXO3
FOXO4
Gabpa
Gata1
GATA1::TAL1
GATA2
GMEB2
HIC2
HLF
HMBOX1
Hoxa9
Id2
IRF4
JDP2
JUNB
JUND
Klf1
Klf12
MAFF
MAFK
MEF2B
MEIS1
MGA
MLX
Mlxip
NFE2
Nfe2l2
NFIL3
NFIX
NFKB1
NFKB2
NRF1
PAX5
PBX3
RARA
Rfx1
RFX2
RFX3
RFX5
RUNX1
RUNX3
SMAD3
SP2
SP4
SPI1
Stat4
Stat6
Tcf7
TFDP1
TFE3
TFEB
USF2
MEP
Erythroid
B Cell
T Cell
CMP
GMP
Monocyte
CMP
GMP
MEP
Monocyte
Erythroid
B Cell
T Cell
B
-log10 (p-value)
30
25
20
15
10
5
0
Cell
Atf1
Atf3
ATF4
BACH2
BHLHE40
CEBPA
CEBPB
CEBPD
Creb5
CUX1
E2F4
EBF1
EGR1
EGR2
ELF1
ELF4
ELK1
ELK3
ELK4
ERF
ERG
Esrra
ETS1
ETV3
ETV6
FLI1
FOS
FOXO3
FOXO4
Gabpa
Gata1
GATA1::TAL1
GATA2
GMEB2
HIC2
HLF
HMBOX1
Hoxa9
Id2
IRF4
JDP2
JUNB
JUND
Klf1
Klf12
MAFF
MAFK
MEF2B
MEIS1
MGA
MLX
Mlxip
NFE2
Nfe2l2
NFIL3
NFIX
NFKB1
NFKB2
NRF1
PAX5
PBX3
RARA
Rfx1
RFX2
RFX3
RFX5
RUNX1
RUNX3
SMAD3
SP2
SP4
SPI1
Stat4
Stat6
Tcf7
TFDP1
TFE3
TFEB
USF2
MEP
Erythroid
B Cell
T Cell
CMP
GMP
Monocyte

Figure S8. Lineage specific enhancers harbour lineage specific TF binding site are marked with H3K27ac in progenitor population. A) Enrichment of Transcription factor binding site at H3K27ac enriched regions with respect to CD34+CD38- compartment. **B)** Significant value for enrichment of TF binding site shown in **(A)**.
