# Supplementary figures and images for: Polycomb contraction differentially regulates terminal human hematopoietic differentiation programs
Source: BMC Biol. 2022 May 13;20:104. doi: 10.1186/s12915-022-01315-1 (PMC9102747; doi:10.1186/s12915-022-01315-1)

Original Gel Image Figure 5D

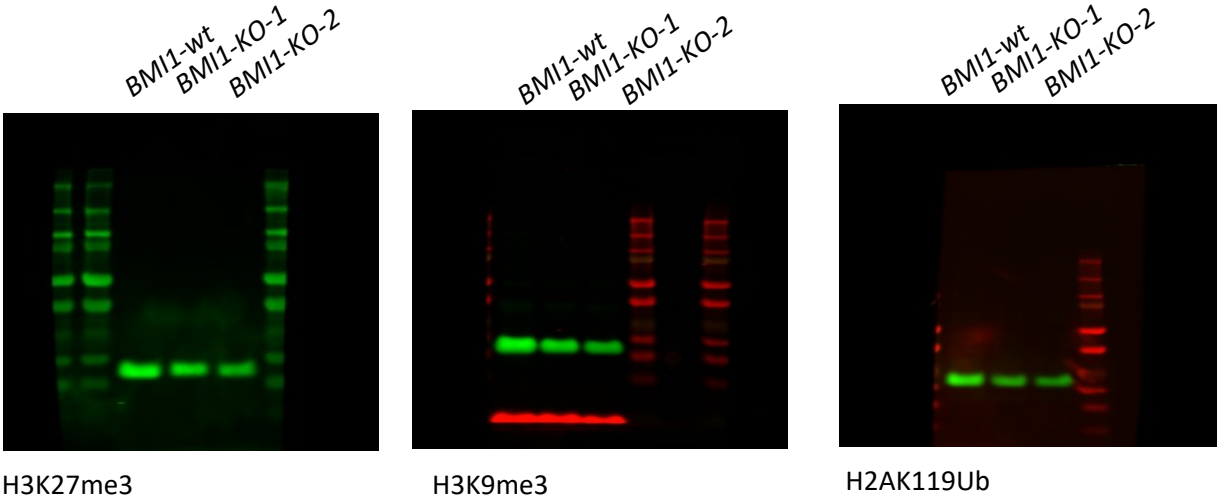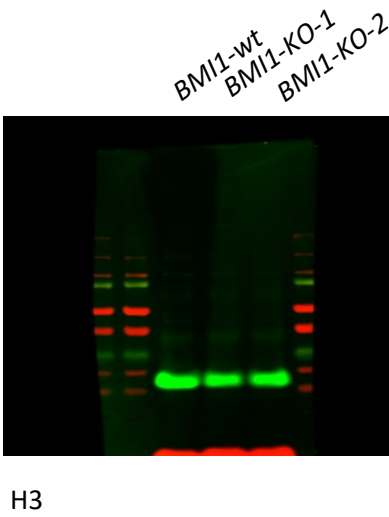

Original Gel Image Figure S5B and E

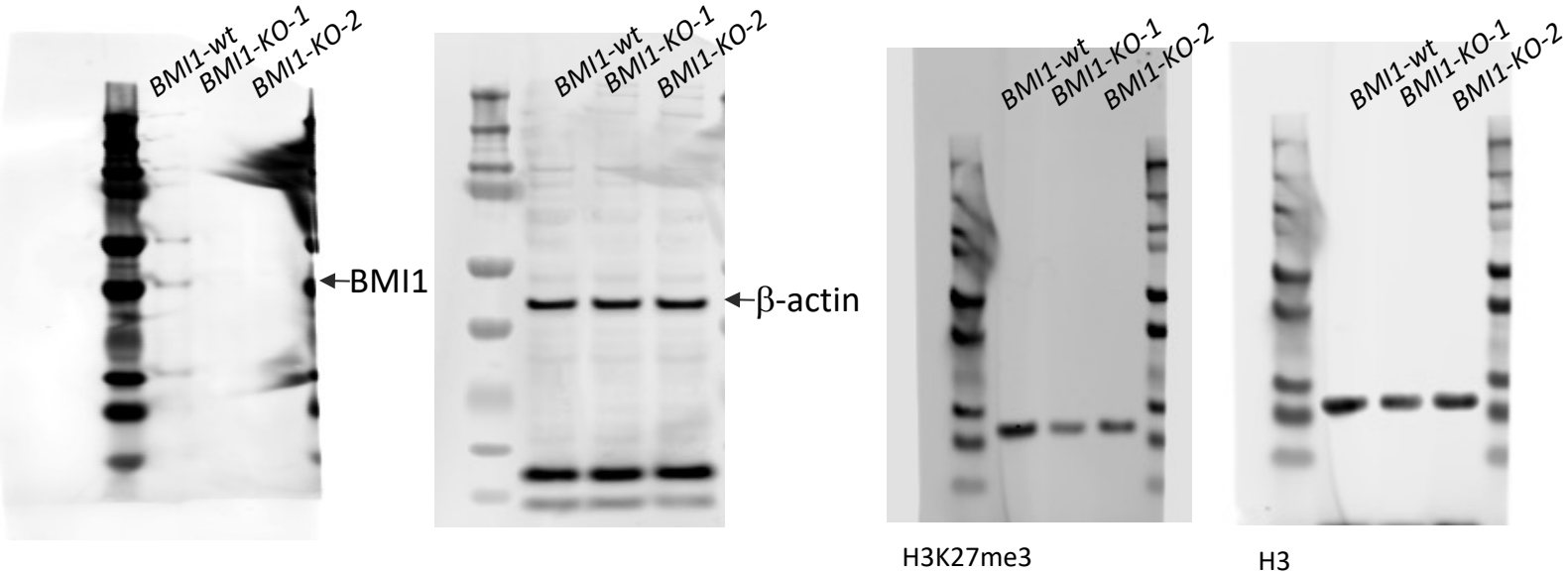

Supplement: Supplementary file 3 — Additional file 3.. Original Gel Images. [file 12915_2022_1315_MOESM3_ESM.pdf]
